# Efficacy of traditional Chinese exercises in improving anthropometric and biochemical indicators in overweight and obese subjects: A systematic review and meta-analysis

**DOI:** 10.1097/MD.0000000000033051

**Published:** 2023-03-24

**Authors:** Qianfang Yang, Fan Wang, Limin Pan, Ting Ye

**Affiliations:** a Heilongjiang University of Traditional Chinese Medicine, Harbin, Heilongjiang Province, China; b Harbin Medical University, Harbin, Heilongjiang Province, China; c The First Affiliated Hospital of Heilongjiang University of Traditional Chinese Medicine, Harbin, Heilongjiang Province, China; d Second Affiliated Hospital of Heilongjiang University of Traditional Chinese Medicine, Harbin, Heilongjiang Province, China.

**Keywords:** anthropometric, biochemical, meta-analysis, obese, traditional Chinese exercise

## Abstract

The efficacy of traditional Chinese exercise (TCE)-based intervention in the improvement of anthropometric and biochemical indicators in overweight and obese patients is controversial. In this regard, the aim of this review was to summarize the evidence of TCE interventions to evaluate their effectiveness on the anthropometric and biochemical indicators of overweight and obese patients.

**Method:**

Five databases were systematically searched for relevant articles published from inception to October 2022. Randomized controlled trials examining TCE intervention in overweight and obese patients The treatment effects were estimated using a random-effect meta-analysis model with standardized mean differences (Hedges’ *g*). The categorical and continuous variables were used to conduct moderator analyses. This review was registered in the International Prospective Register of Systematic Reviews (PROSPERO) (identifier CRD42022377632).

**Result:**

Nine studies involving a total of 1297 participants were included in the final analysis. In the anthropometric indicators outcomes, the meta-analytic findings revealed large and significant improvements in body mass index (*g* = 1.44, 95% confidence interval [CI] = 1.27–1.61, *P* = .000, *I*^2^ = 99%), weight (*g* = 1.47, 95% CI = 1.25–1.68, *P* = .000, *I*^2^ = 95%), fat percentage (*g* = 1.22, 95% CI = 0.52–1.93, *P* = .000, *I*^2^ = 93%), and small and significant improvements in waist circumference (*g* = 0.38, 95% CI = 0.21–0.54, *P* = .000, *I*^2^ = 99%). In the biochemical indicators outcomes, the findings revealed large and significant improvements in low density lipoprotein (*g* = 2.08, 95% CI = 1.80–2.37, *P* = .000, *I*^2^ = 98%), moderate and significant improvements in triglyceride (*g* = 0.69, 95% CI = 0.56–0.81, *P* = .000, *I*^2^ = 96%), small and significant improvements in total cholesterol (*g* = 0.37, 95% CI = 0.19–0.54, *P* = .000, *I*^2^ = 77%), and high-density lipoprotein (*g* = −0.71, 95% CI = −0.86 to 0.57, *P* = .000, *I*^2^ = 99%). The moderator shows that the effects of TCE on anthropometric and biochemical indicators were moderated by frequency of exercise, exercise duration, and type of control group.

**Conclusion:**

TCE intervention is a beneficial non-pharmacological approach to improving anthropometric and biochemical indicators in overweight and obese subjects, especially in body mass index, weight, fat percentage, triglyceride, and low-density lipoprotein. The clinical relevance of our findings is pending more extensive trials and more rigorous study designs to strengthen the evidence.

## 1. Introduction

Being overweight or obese is a chronic nutritional disorder that causes excessive accumulation of body fat due to a combination of genetics, environment, and dietary behaviors.^[1,2]^ The World Health Organization (WHO) defines “overweight” as a body mass index (BMI) of 25.0 kg/m^2^ to 29.9 kg/m^2^, and “obesity” as a BMI of 30.0 kg/m^2^. But, there is currently no international standard that applies to all countries or regions; for example, the WHO defines “overweight” as a BMI of 23 kg/m^2^ to 27.5 kg/m^2^ for Chinese and a BMI of 27.5 kg/m^2^ for the “obese” group.^[3,4]^ The global prevalence of overweight or obesity has doubled since 1980, affecting approximately 1 to 3rd of the population.^[5]^

The occurrence and progression of overweight or obesity are closely related to cardiovascular disease, dyslipidemia, and insulin resistance, which in turn lead to a variety of comorbidities and chronic diseases, such as diabetes, stroke, gallstones, fatty liver, etc. In terms of treatment, clinically individualized treatment is adopted, starting from the cause, and includes dietary modification, behavioral intervention, drug treatment, and surgical treatment if necessary.^[6]^ In addition, the active ingredients in many natural plants play an important role in the treatment of obesity. For example, phenolic acids and polyphenols contained in natural plants of Lamiaceae and Rosaceae have been widely used in anti-obesity treatment.^[7]^

However, despite multiple treatment modalities, anthropometric and biochemical markers remain problematic in patients who are overweight or obese, often leading to the development of other comorbidities with serious consequences.^[8]^Experts recommend exercise and dietary modification therapy as an effective adjunct to the treatment of overweight or obesity because of deficient drug management and practices for overweight or obesity.^[9,10]^In a study investigating the effects of aerobic exercise on body composition in overweight and obese women, significant differences in body composition were found among participants in the training group.^[11]^Physical activity increases energy expenditure and improves resting metabolic rate, cardiopulmonary function, and the physical component in overweight or obese patients.^[12]^ In recent years, traditional Chinese exercise (TCE) have had significant advantages over medicine and surgery in treating overweight or obesity. Taijiquan and Qigong (Baduanjin, Yijinjing, Wuqinxi, etc), as China’s national intangible cultural heritage,^[13]^ are forms of movement in Eastern culture; they are both internal and external, resilient and soft, and are being welcomed by more and more people.

Studies have found that tai chi exercise can gradually restore the normal activity of abnormally expressed AMPK genes in obese patients and significantly reduce triglycerides, total cholesterol, and low density lipoprotein (LDL) cholesterol indicators. In addition, it can also reduce fasting blood sugar, improve the microinflammatory state caused by obesity, and have a good balancing effect on the body’s energy metabolism.^[14–17]^ However, some randomized controlled trials (RCTs) on the health benefits of TCE in people who are overweight or obese have yielded inconclusive results. In the effect of adding Tai Chi to dietary weight loss programs on lipoprotein hardness in obese older women,^[18]^ for example, neither the Tai Chi nor the diet education groups improved their LDL cholesterol. Another study^[19]^showed that tai chi is a low-intensity physical exercise that consumes less energy and is detrimental to beneficial changes in body composition. In a study of the combined effects of tai chi, resistance training, and diet on body function and body composition in obese older women, Maris^[20]^ found that the Tai Chi group was more helpful than the control group for improving physical function (e.g., TUG time), but there was no significant increase in body composition or functional indicators.

To date, there have been no meta-analyses to assess whether various types of TCEs (Tai Chi, Qigong, Baduan Jin, Yijin jing, Wuqinxi, etc) and exercise frequency, duration, and number of sessions have an effect on overweight or obese patients. Furthermore, none of them computed effect sizes using Hedges’ g-statistic. Other comprehensive studies are necessary to confirm the effect of TCE on anthropometric and biochemical markers in overweight or obese patients. Therefore, the main objective of this study is to determine the effect of traditional Chinese exercise on anthropometric and biochemical indicators in overweight or obese patients. The second objective was to determine whether any underlying moderating factors (e.g., control type) and TCE dose-related variables (e.g., frequency of exercise, duration of exercise, and number of sessions) affected the effect of the intervention.

## 2. Methods

The results of this meta-analysis are reported in accordance with the Preferred Reporting Items for Systematic Reviews and Meta-Analysis (PRISMA) guidelines.^[21]^

### 2.1. Search strategy

First, we searched the Pubmed, Cochrane Library, EMBASE, Web of Science, and Scopus databases from inception to October 2022. Second, the reference lists of the included studies were examined as an additional check for potential studies that could be used in this review. The following keywords were used: “Traditional Chinese exercise” or “Tai Chi” or “Qigong” or “BaduanJin” or “Wuqinxi” or “Yijinjing”; AND; “Obesity” or “obesity disease”; AND; “Randomized controlled trials” or “clinical trials.” The specific search syntax, such as PubMed, can be found in the Supplemental Digital Content (File “Search,” http://links.lww.com/MD/I524).

### 2.2. Eligibility criteria

Eligibility criteria were formulated based on the PICOS framework.

1. Participants: Participants who were overweight or obese [BMI > 23 kg/m^2^ or waist circumference 90 cm (men) or 80 cm (women)].
2. Intervention: An experimental group is included in the form of TCE (Tai chi, qigong, etc).
3. Control: The comparison group received either an alternative intervention (walking, dancing, etc) or no intervention. Alternative interventions included routine exercise and health education but did not include TCE activities.
4. Outcome: Anthropometric indicators and biochemical indicators were evaluated as the outcomes, and all included outcome indicators were assessed at the beginning and end of the intervention.
5. Study: Eligible studies were peer-reviewed articles reporting results from RCTs that examined the effects of TCE on anthropometric and biochemical indicators in overweight or obese participants. The following types of articles were excluded: Prospective or retrospective cohort studies; Case reports; Conference abstracts; and Articles not written in English.

### 2.3. Study selection

Two reviewers (QY and FW) conducted the initial online search independently to avoid selection bias. After excluding duplicate studies, article titles and abstracts were reviewed. If an abstract was considered relevant or ambiguous, the full text was reviewed, and inclusion and exclusion criteria were applied. The Kappa statistic was used to assess the reliability of data selection and selection between 2 reviewers (QY and FW). Cohen suggested the Kappa result be interpreted as follows: values ≤ 0 as indicating no agreement and 0.01 to 0.20 as none to slight, 0.21 to 0.40 as fair, 0.41 to 0.60 as moderate, 0.61 to 0.80 as substantial, and 0.81 to 1.00 as almost perfect agreement.^[22]^

### 2.4. Data extraction and statistical analysis

Two reviewers (QY and FW) independently evaluated the article and extracted the data. The details of the retrieved articles are summarized in Table 1. Study feature data were extracted for each article (first author; year of publication; country; experimental design; average age; proportion of females; sample size; average BMI; usual care during the experiment; intervention characteristics, including type, frequency, and duration of intervention). Passive intervention was defined as a blank control group in the control group, while the exact total training time in the experimental group was defined as active intervention. During the data extraction process, any conflicts or ambiguities in the reporting method or results will be discussed with a third reviewer (TY) and resolved by consensus.

**Table 1:** Characteristics of randomized controlled trials included in the meta-analysis.

References	Country	Study design	Participant characteristics					Intervention protocol		Outcome	Adverse effects
			Age, mean (SD)	%Sex Female, (EG/CG)	N (EG/CG)	BMI, mean (SD)	C	Intervention	Control		
Liu et al (2015)	Australia	RCT	EG: 52 (12) CG: 53 (11)	75/74	213 (106/107)	EG: 34.8 (6.6) CG: 35.1 (7.1)	Depression	Tai chi 3 × 90 min/wk 24 wk	No intervention	BMI; WC; TG; HDL	Heart failure (n = 1) Depression (n = 1)
Chen et al (2010)	China	RCT	EG: 59.1 (6.2) CG: 57.4 (5.8)	55.4/58.3	104 (56/48)	EG: 33.5 (4.7) CG: 33.2 (4.1)	Type 2 diabetes	Tai chi 3 × 60 min/wk 12 wk	Aerobic exercises	BMI; TG; TC; HDL	NR
Sun et al (2015)	China	RCT	45-64 and ≥ 65	86/78	266 (136/130)	EG: 23.38 (3.05) CG: 23.50 (2.99)	Hypertension	Tai chi 3 × 60 min/wk 2 mo	Reading and learning computer software	BMI; WC; TC; HDL; LDL; TG	NR
Beebe et al (2013)	USA	RCT	EG: 60.4 (6.2) CG: 62.6 (5.9)	100/100	26 (13/13)	EG: 33.7 (4.8) CG: 34.8 (2.9)	Coronary heart disease	Tai chi 3 × 60 min/wk 16 wk + diet education	Diet education	BMI; W; WC; HDL; LDL; TC; TG	NR
Siu et al (2021)	China	RCT	EG: 62.6 (6.2) CG: 61.0 (5.7)	77.3/79.0	543 (181/181/181)	EG: 25.5 (3.6) CG: 25.5 (3.4)	NR	Tai chi 3 × 60 min/wk 12 wk	UC	BMI; W; WC; HDL; TG	NR
Dechamps et al (2009)	France	RCT	44.4 (11.9)	100/100	21 (11/10)	EG: 37.4 (4.8) CG: 38.5 (7.3)	NR	Tai chi 2 × 60 min/wk 10 wk	Physical activity	BMI; W; FP	NR
Leung et al (2019)	China	RCT	EG: 62.19 (5.93) CG: 65.52 (9.34)	41/56	54 (27/27)	EG: 27.40 (4.82) CG: 27.25 (4.34)	Metabolic syndrome	Tai chi 2 × 60 min/wk 12 wk + Tai chi 3 × 30 min/wk 12 wk	Non-exercise recreational class	WC; TC; TG; HDL	NR
Soltero et al (2022)	USA	RCT	EG: 49.6 (6.22) CG: 53.2 (9.30)	100/100	20 (10/10)	EG: 29.48 (0.90) CG:31.14 (1.18)	Breast cancer	Qigong/Tai Chi 2 × 60 min/wk 8 wk	Latin dance	BMI; FP	NR
Li et al (2022)	China	RCT	EG: 23.2 (4.38) CG:22.9 (4.64)	100/100	50 (30/20)	EG: 28.41 (4.03) CG:29.57 (4.47)	Polycystic ovary	Tai chi 3 × 60 min/wk 12 wk	Routine exercise	BMI; W; TG; TC; HDL; LDL	NR

BMI = body mass index, C = complications, CG = control group, EG = experimental group, FP = fat percentage, HDL = high density lipoprotein, LDL = low density lipoprotein, NR = no report, RCT = randomized controlled trial, SD = standard deviation, TC = total cholesterol, TG = triglyceride, W = weight, WC = waist circumference.

All data was entered as the mean with standard deviation for the TCE and control groups at baseline and immediately after training. If the data was unsuitable for our analysis, the previous statistical formula was used to convert the data into mean and standard deviation format.^[23]^All analyses were conducted using comprehensive meta-analysis Version 3.3 software (Biostat Inc., Englewood, NJ). The comprehensive meta-analysis allows for each of these different study outcomes to be flexibly entered into the model. A random effects model was used to correct for variable effect sizes across the studies if these studies showed heterogeneity in their intervention. We used an inter group, pre-to-post-intervention, meta-analysis design based on standardized mean differences (Hedges’ *g*). Hedges’ *g*, a variant of Cohen d that corrects for sample size biases, was used to calculate the effect sizes (ESs).^[24]^We chose the Hedges’ *g* of the ESs to estimate the efficacy of TCE intervention. Hedge g estimates of < 0.3 were considered as small, ≥0.3 and < 0.6 as moderate, and ≥ 0.6 as large, respectively.^[25]^ Positive effect sizes indicated a more favorable outcome for the experimental group. The *I*^2^ statistic estimated heterogeneity among studies and classified it as 25% (low heterogeneity), 50% (moderate heterogeneity), or 75% (high heterogeneity).^[24]^ A sensitivity analysis was also performed to detect the presence of highly influential studies that could skew the results. Studies were deemed influential if their removal significantly modified the summary effect. In addition, a moderator analysis was conducted based on exercise frequency, exercise duration, number of sessions, and type of control group. The significance level was set at *P ≤* .05.

### 2.5. Risk of bias and study quality assessment

**Figure 1.**
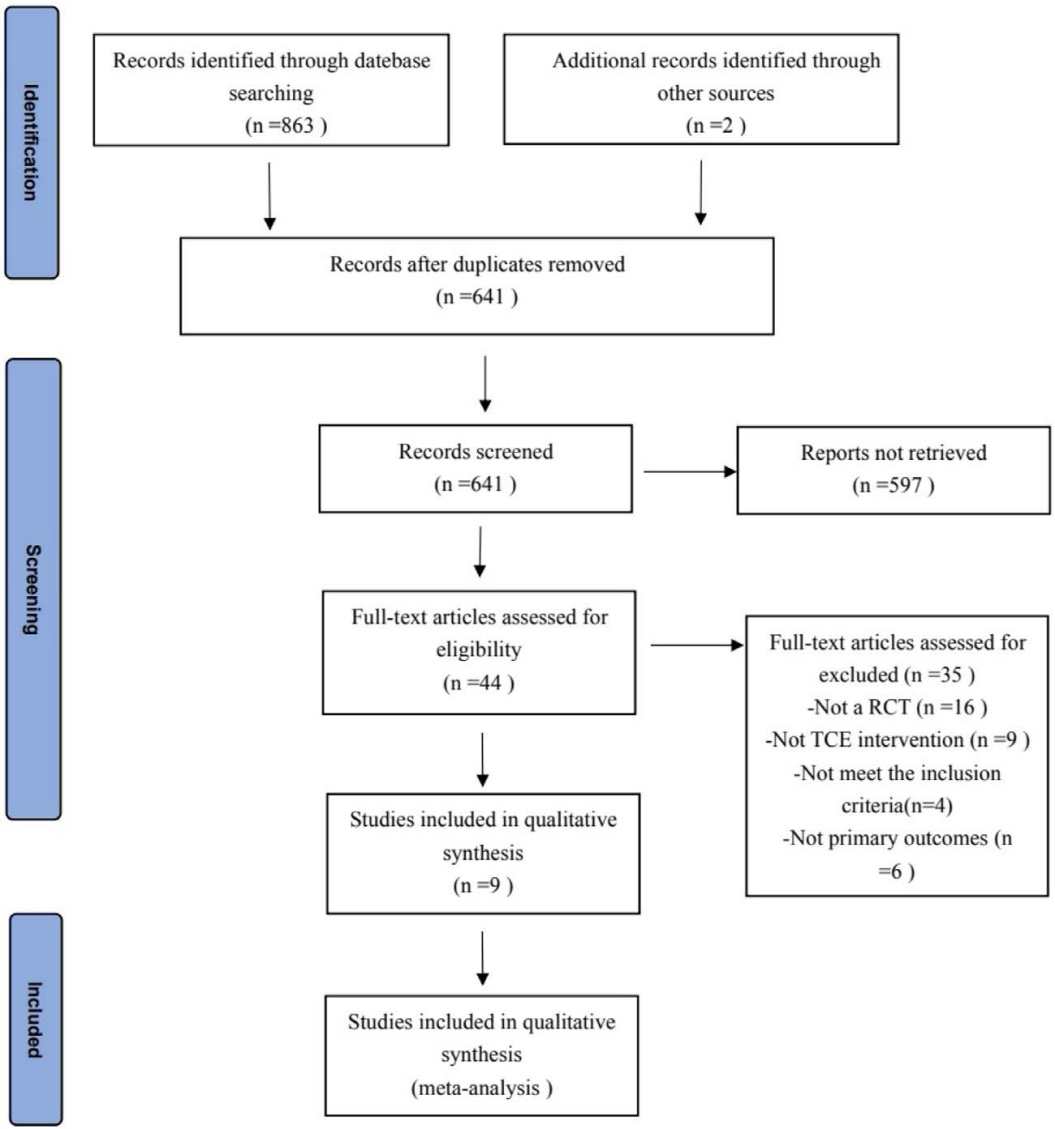
Assessment of risk of bias with selected studies.

The Cochrane Collaboration’s tool was used to assess the risk of bias in each individual study.^[26]^ The tool contains the domains of sequence generation, allocation concealment, blinding of participants, personnel, and outcome assessors, incomplete outcome data, selective outcome reporting, and other sources of bias. We classified items as “low risk,” “high risk,” or “unclear risk” of bias. The risk of bias is presented in Figure 1.

We used the PEDro scale (Physiotherapy Evidence Database Rating Scale) to assess the quality of included studies using a score (Table 2).^[27]^ The scale includes 11 items to rate study quality, and the maximum score is 11. Studies that scored 7 or higher were considered high quality, while those that scored 6 or lower were considered low quality. The scoring process was conducted by 2 authors (QY and FW). TY established consensus scores and resolved any disagreements.

**Table 2:** Physiotherapy evidence database (PEDro) scores of the 9 included studies.

References	PEDro scores	Methodological quality	PEDro item number										
			1	2	3	4	5	6	7	8	9	10	11
Liu et al (2015)	8	H	1	1	1	1	0	0	1	0	1	1	1
Chen et al (2010)	8	H	1	1	1	1	0	0	0	1	1	1	1
Sun et al (2015)	9	H	1	1	1	1	0	0	1	1	1	1	1
Beebe et al (2013)	8	H	1	1	1	1	0	0	0	1	1	1	1
Siu et al (2021)	9	H	1	1	1	1	0	0	1	1	1	1	1
Dechamps et al (2009)	9	H	1	1	1	1	0	0	1	1	1	1	1
Leung et al (2019)	8	H	1	1	1	1	0	0	1	0	1	1	1
Soltero et al (2022)	6	L	1	1	0	1	0	0	0	0	1	1	1
Li et al (2022)	8	H	1	1	1	1	0	0	1	0	1	1	1

Studies were classified as having high quality (≥7), low quality (≤6). 0 = does not meet the criteria; 1 = meets the criteria. Criteria (without eligibility criteria) were used to calculate the total PEDro score; Item 1 = Inclusion conditions; Item 2 = Random distribuion; Item 3 = Allocation hiding; Item 4 = Baseline similarity; Item 5 = Subjects blinded; Item 6 = Therapist blinding; Item 7 = Assessor blinding; Item 8 = Primary outcome measures (Subjects, ≥85%); Item 9 = Intention-to-treat analysis; Item 10 = Inter group statistical results report; Item 11 = Point measurements and variation measurements.

H = high quality, L = low quality.

### 2.6. Publication bias

We use Stata 17.0 software for Egger test and Begg test. The Egger test and Begg test were used to identify publication bias. If the 95% confidence interval of the intercept of the regression equation is found to contain 0 and *P* > .05, it indicates unbiased; otherwise, it is biased.

## 3. Results

### 3.1. Search results

**Figure 2.**
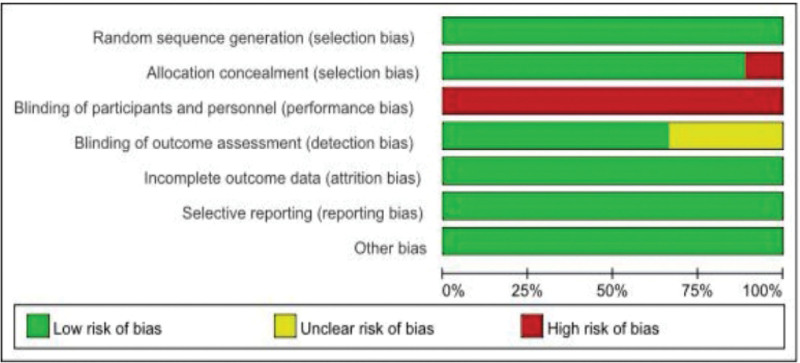
Process of study selection following the preferred reporting items for systematic reviews and meta-analyses (PRISMA).

Figure 2 shows the flow diagram of study selection. The initial search yielded 863 articles from 7 databases, and 2 additional articles were identified through other sources. After removing duplicates, 641 eligible records were retrieved. After reading the titles and abstracts, 597 articles were excluded. After reading the full text, the remaining 44 articles met the inclusion criteria. Among these 44 articles, 16 were excluded because the study design was not an RCT, 9 because TCE was not used as the intervention, 4 because participants did not meet the inclusion criteria, and 6 because the outcome measures were not the primary outcome. Finally, 9 original research articles were selected for further analysis.^[28–36]^

### 3.2. Study characteristics

Table 1 lists the characteristics of each of the included studies, which were published between 2009 and 2022. Five studies were conducted in China^[29,30,32,34,36]^; 2 in USA^[31,35]^; 1 in France^[33]^; 1 in Australia.^[28]^ The TCE program was used to treat overweight or obese participants in all experimental groups. Active (routine exercise, diet education, etc) or passive interventions (nonintervention) were employed in the control group. These participants were prescribed 60 to 90 minutes of exercise in each session 2 to 3 times per-week for 8 weeks to 12 months. The outcomes of these 9 studies were as follows: body mass index (BMI), waist circumference (WC), weight (W), fat percentage (FP), total cholesterol (TC), triglyceride (TG), high density lipoprotein (HDL), and LDL. One study^[28]^reported on suspected side effects such as heart failure (n = 1) and depression (n = 1). The remaining studies reported no TCE related side effects.

### 3.3. Synthetic results

**Figure 3.**
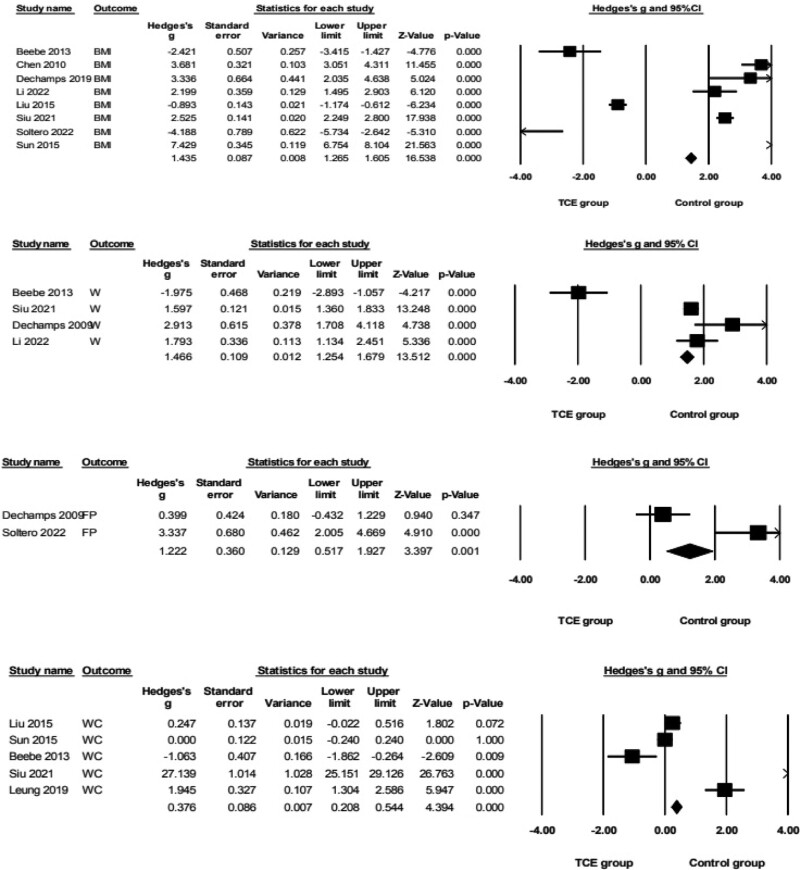
Forest plot showing the effects of TCE vs control group on anthropometric indicators outcomes: BMI, WC, W, FP. BMI = body mass index, FP = fat percentage, TCE = traditional Chinese exercise, W = weight, WC = waist circumference.

Regarding anthropometric indicators outcomes (Fig. 3), the pooled data demonstrated that TCE resulted produced large and significant improvements in BMI (*g* = 1.44, 95% confidence interval [CI] = 1.27–1.61, *P* = .000, *I*^2^ = 99%), W (*g* = 1.47, 95% CI = 1.25–1.68, *P* = .000, *I*^2^ = 95%) and FP (*g* = 1.22, 95% CI = 0.52–1.93, *P* = .000, *I*^2^ = 93%) when compared to the control group. Moreover, pooled analyses from 5 parallel trials^[28,30–32,34]^revealed that the WC exerted a small and significant increase in effect size (*g* = 0.38, 95% CI = 0.21–0.54, *P* = .000, *I*^2^ = 99%) compared with the control group.

**Figure 4.**
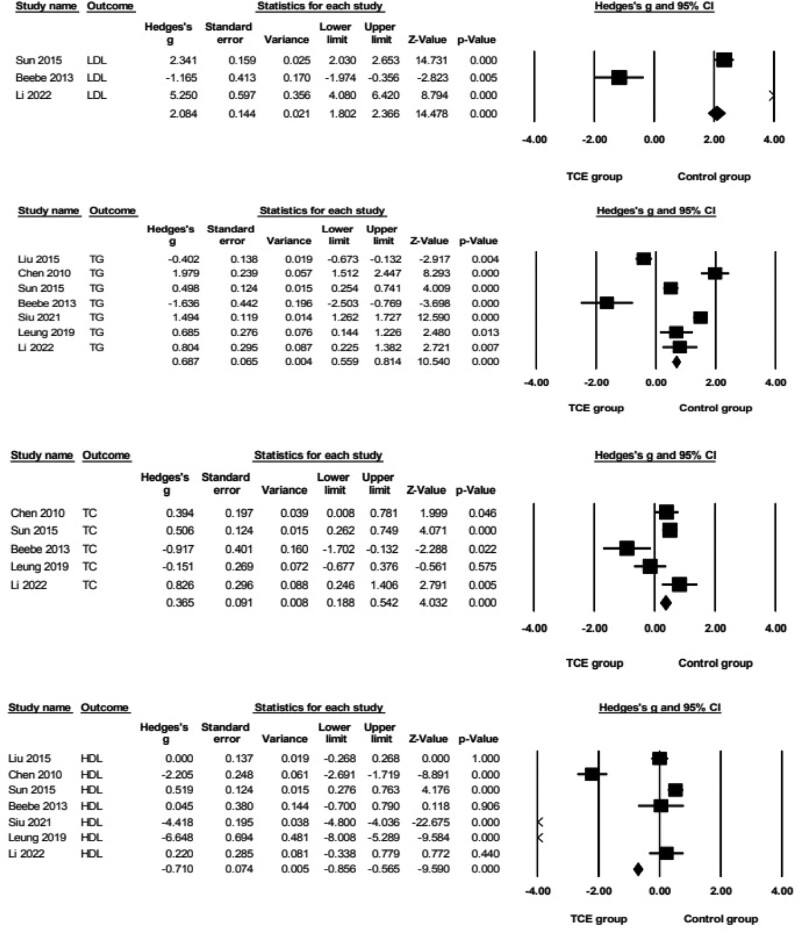
Forest plot showing the effects of TCE vs control group on biochemical indicators outcomes: LDL, TG, TC, and HDL. HDL = high density lipoprotein, LDL = low density lipoprotein, TC = total cholesterol, TCE = traditional Chinese exercise, TG = triglyceride.

Regarding biochemical indicators outcomes (Fig. 4), the pooled data demonstrated that TCE resulted produced large and significant improvements in LDL (*g* = 2.08, 95% CI = 1.80–2.37, *P* = .000, *I*^2^ = 98%) when compared to the control group. Pooled analyses from 7 parallel trials^[28–32,34,36]^revealed that the TG exerted a moderate and significant increase in effect size (*g* = 0.69, 95% CI = 0.56–0.81, *P* = .000, *I*^2^ = 96%) compared with the control group. Moreover, the pooled data demonstrated that TCE resulted produced small and significant improvements in TC (*g* = 0.37, 95% CI = 0.19–0.54, *P* = .000, *I*^2^ = 77%) and HDL (*g* = −0.71, 95% CI = −0.86 to 0.57, *P* = .000, *I*^2^ = 99%) when compared to the control group.

According to the sensitivity analysis, no study significantly impacted the outcomes. No study was deemed insignificant since its removal had no discernible effect on the overall effect.

### 3.4. Moderator analysis

The categorical and continuous variables in Table 3 were used to conduct moderator analyses. In terms of frequency of exercise, more than 3 sessions/week (*g* = 1.47, 95% CI = 1.30–1.64, *P* = .000) of TCE large and significant improved BMI compared with <3 sessions/week (*g* = 0.22,95% CI = −0.78 to 1.21, *P* = .673). Additionally, more than 3 sessions/week RCTs (*g* = 0.43, 95% CI = 0.24–0.62, *P* = .000) of TCE small and significantly improved TC compared with less than 3 sessions/week RCTs on TCE (*g* = −0.15, 95% CI = −0.68 to 0.38, *P* = .575). In terms of type of control group, compared to RCTs on TCE with non-active control group (*g* = −0.89, 95% CI = −1.17 to 0.61, *P* = .000), RCTs on TCE with active control group (*g* = 2.78, 95% CI = 2.57–3, *P* = .000) significantly improved BMI. Moreover, active control group RCTs (*g* = 1.00, 95% CI = 0.85–1.14, *P* = .000) of TCE large and significantly improved TG compared with non-active control group RCTs on TCE (*g* = −0.4, 95% CI = −0.67 to 0.13, *P* = .004).

**Table 3:** Moderator analysis for the effects of TCE on measurement outcomes.

Variables	Anthropometric indicators outcomes				Biochemical indicators outcomes			
	BMI Hedges’ *g* (95% CI)	WC Hedges’ *g* (95% CI)	W Hedges’ *g* (95% CI)	FP Hedges’ *g* (95% CI)	TC Hedges’ *g* (95% CI)	TG Hedges’ *g* (95% CI)	HDL Hedges’ *g* (95% CI)	LDL Hedges’ *g* (95% CI)
Exercise frequency								
≥3sessions/wk	1.47 (1.30 to 1.64)	0.26 (0.09 to 0.44)	1.42 (1.2 to 1.64)	–	0.43 (0.24 to 0.62)	0.69 (0.56 to 0.82)	−0.64 (−0.79 to −0.5)	2.08 (1.8–2.37)
<3sessions/wk	0.22 (−0.78 to 1.21)	1.95 (1.3 to 2.59)	2.91 (1.71 to 4.12)	1.22 (0.52–1.93)	−0.15 (−0.68 to 0.38)	0.69 (0.14 to 1.23)	-6.65 (−8.01 to −5.29)	–
Exercise duration								
>12 wk	0.16 (−0.09 to 0.41)	0.05 (−0.12 to −0.23)	−1.98 (−2.89 to −1.06)	–	0.38 (0.15 to 0.61)	0.02 (−0.15 to 0.20)	0.27 (0.10 to 0.47)	1.89 (1.60–2.18)
≤12 wk	2.52 (2.29 to 2.75)	4.32 (3.71 to 4.93)	1.66 (1.44 to 1.88)	1.22 (0.52–1.93)	0.34 (0.07 to 0.62)	1.41 (1.22 to 1.6)	−2.87 (−3.13 to −2.61)	5.25 (4.08–6.42)
Number of sessions								
>36 sessions	0.16 (−0.09 to 0.41)	0.18 (0.02 to 0.35)	−1.98 (−2.89 to −1.06)	–	0.29 (0.08 to 0.51)	0.09 (−0.08 to 0.26)	0.16 (−0.02 to 0.33)	1.89 (1.60–2.18)
≤36 sessions	2.52 (2.29 to 2.75)	27.14 (25.15 to 29.13)	1.66 (1.44 to 1.88)	1.22 (0.52–1.93)	0.53 (0.21 to 0.85)	1.5 (1.30 to 1.70)	−2.72 (−2.99 to −2.46)	5.25 (4.08–6.42)
Type of control group								
Active control	2.78 (2.57 to 3.00)	0.46 (0.24 to 0.67)	1.47 (1.25 to 1.68)	1.22 (0.52–1.93)	0.37 (0.19 to 0.54)	1.00 (0.85 to 1.14)	−1.01 (−1.18 to −0.83)	2.08 (1.8–2.37)
Non-active control	−0.89 (−1.17 to −0.61)	0.25 (−0.02 to 0.52)	to	–	to	−0.4 (−0.67 to −0.13)	0.00 (−0.27 to 0.27)	–

BMI = body mass index, FP = fat percentage, HDL = high density lipoprotein, LDL = low density lipoprotein, TC = total cholesterol, TCE = traditional Chinese exercise, TG = triglyceride, W =weight, WC = waist circumference

### 3.5. Risk of bias and study quality

Figure 1 show the risk of bias in the 9 included studies. All studies used random sequence generation. In the term of allocation concealment, 8 studies^[28–34,36]^ had a low risk of attrition bias and 1 study had a high risk of attrition bias. In the term of blinding of outcome assessors, 6 studies^[28,30,32–34,36]^ had a low risk of attrition bias and 3 studies^[29,31,35]^ had a unclear risk of attrition bias. Moreover, all studies had a high risk of attrition bias on blinding of therapist and participants. Additionally, all studies had a low risk of attrition bias on incomplete outcome data, incomplete outcome data and other sources of bias. The value of Kappa calculated for the various parameters extracted by the 2 investigators was 0.83 (*P* < .001), indicating an excellent degree of inter-investigator agreement.

Table 2 presents the methodology quality of the included studies. The quality of the studies ranged between high quality and low quality (score range: 6–9 points), with 8 studies being classified as high quality^[28–34,36]^and 1 study as low quality.^[35]^ Eight studies used a concealed allocation procedure^[28–34,36]^ and all reported random assignment. We could not blind patients and therapists because this was an interventional movement study. However, 6 trials blinded the outcome assessors.^[28,30,32–34,36]^ Five studies had a dropout rate of > 85%.^[29–33]^ All 9 studies performed intention-to-treat analyses, between-group statistics, and point measurements.

### 3.6. Publication bias

The Egger test and Begg test (Figure S1–S8, Supplemental Digital Content, http://links.lww.com/MD/I525) were used to assess publication bias. The results of the Egger test are as follows: BMI (*P* = .3>0.05), WC (*P* = .18>0.05), TC (*P* = .27>0.05), TG (*P* = .76>0.05) all showed no publication bias. The results of the Begg test are as follows: BMI (*P* = .23>0.05), WC (*P* = .22>0.05), TC (*P* = .46>0.05), TG (*P* = .55>0.05) all showed no publication bias.

## 4. Discussion

The current systematic review with meta-analysis showed that TCE significantly improved anthropometric indicator outcomes (BMI, WC, W, and FP), biochemical indicators outcomes (TC, TG and LDL) compared with the control group in individuals with overweight and obese. However, TCE interventions did not have a significant effect on HDL. Data from 9 RCTs involving a total of 1297 participants were analyzed. The moderator analyses showed the effects of TCE on anthropometric and biochemical indicators were moderated by the sample size, type of control group, frequency of exercise, and exercise duration.

### 4.1. Gaps in previous reviews

To our knowledge, this is the first meta-analysis to focus on the effects of TCE interventions on anthropometric and biochemical indicators in overweight or obese patients. The overall conclusions of our study were similar to those reported in other recent reviews, but the analytical methods used in our study differed except for the inclusion of updated evidence. As an example, previous reviews have been limited to qualitative synthesis and have not included quantitative meta-analysis methods.^[37,38]^ Of the studies included in the meta-analysis, 1 meta-analysis was limited to tai chi exercises (no qigong) and the sample size of the study was small, resulting in wide confidence intervals and reduced accuracy.^[39]^ In another meta-analysis, the outcome was too simplistic, assessing only body composition outcomes and not mentioning other measures.^[40]^ Finally, the 2 meta-analyses included in 2020 and 2016 used a broader range of inclusion criteria, combining results from multiple mind-body modalities, including multiple exercise interventions such as martial arts practice, kung fu practice, and tai chi practice.^[41,42]^

### 4.2. Effects of TCE on anthropometric indicators outcomes

At present, traditional anthropometric indicators related to overweight or obesity include height, body mass, waist circumference, and BMI.^[43,44]^ It is well known that BMI can distinguish between overweight or obesity in general, but not body fat content,^[45]^ and WC and FP are effective indicators for measuring visceral fat distribution,^[46,47]^ so WC and FP were used as auxiliary diagnoses in overweight or obese patients. Asians with the same BMI and WC levels may have higher body fat percentages than Western populations due to racial differences in physical characteristics, and BMI or WC values vary by country to race, as in the WHO-recommended diagnostic criteria for overweight or obesity.^[48–50]^ The current study found that TCE could help overweight or obese patients to improve their anthropometric indicators. In 1 study, tai chi exercise was beneficial in regulating physical and mental state in patients with simple obesity, with significant improvements in body fat percentage and body mass index compared with before treatment.^[51]^ In a community study of obese elderly women, significant improvements in body fat mass and waist circumference were found in the tai chi group compared with the control group, and that tai chi exercise intervention may be an effective strategy to improve physical function and risk of coronary heart disease in older adults.^[52]^

### 4.3. Effects of TCE on biochemical indicators outcomes

Dyslipidemia is associated with obesity due to excessive fat accumulation, and the intensity of work stress, unhealthy lifestyle, and diet all directly contribute to excessive fat accumulation.^[3,53]^ Moderate or central obesity subjects have been found to have higher levels of TC, TG, LDL-C, and lower levels of HDL-C compared with nonobese individuals, which may be associated with excess visceral fat in the abdomen.^[54,55]^ There is a strong correlation between overweight or obesity and cardiovascular events and dyslipidemia, and effective control of lipid levels is expected to reduce the incidence of metabolic morbidity and death.^[56,57]^

Concerning the biochemical indicators outcomes, our meta-analysis showed large significant improvement in LDL, moderate significant improvement in TG, small significant improvement in TC and HDL of TCE intervention on the biochemical indicators outcomes. According to a meta-analysis of the effect of tai chi exercise on blood lipid profile, Tai chi may be beneficial for lipid profiles in different age groups and populations. Especially for HDL-C with a potential positive effect.^[39]^ According to our meta-analysis, HDL levels in overweight or obese patients did a small improve after TCE intervention, which is differs from the results of the review described above. In contrast, our findings provide more convincing evidence for a variety of reasons. First, all but one of the included studies had high-quality methodologies according to the PEDro scale tool. However, none of Chau literature quality is mentioned. Second, although we included 9 articles, 9 were related to obesity, and Chau included 20 articles, but only 3 were related to obesity. Therefore, more research is needed on changes in HDL levels of TCE interventions in overweight or obese patients.

### 4.4. Effects of TCE on moderator analyses

Our moderator analysis revealed that the effect of TCE on BMI was significantly higher (*g* = 1.55) when only large sample size RCTs were analyzed, but not when small sample size RCTs were analyzed (*g* = 0.54). Additionally, large sample size RCTs (*g* = 0.74) have a greater effect on TG than small sample size RCTs (*g* = 0.33). Moreover, large sample size RCTs (*g* = 2.34) have a greater effect on LDL than small sample size RCTs (*g* = 0.91). As a result, our findings imply that the significant effects of TCE on BMI, TG, and LDL were not based on small sample size RCTs; therefore, future studies must utilize large sample size RCTs to evaluate the effects of TCE on BMI, TG and LDL in overweight or obese patients. As for the frequency of exercise, BMI and TC improved significantly in overweight or obese patients after they performed more than 3 sessions/week (BMI: g = 1.47; TC: g = 0.27) compared to less than 3 sessions/week (BMI: g = 0.22; TC: g = −0.15). Moderator analysis showed that TCE increased the effect on active control group (BMI: g = 2.78; TG: g = 1.00) compared with non-active control group (BMI: g = −0.89; TG: g = −0.4).

### 4.5. Strengths and limitations

The strength of this meta-analysis is that our systematic review and meta-analysis followed the PRISMA statement to the letter, and our review methodology was registered. Only the RCT design was chosen due to its reliability. Furthermore, we used Hedges’ g to ensure an accurate estimation of the overall effect size. Other potential confounding variables were explored to determine their impact on the effects of TCE. However, there are limitations in this study. First, for the included RCTs, although the literature was searched and screened in strict accordance with the search strategy and inclusion criteria, there was a certain degree of missed detection and bias. Second, the number of RCTs included in this study was 9, and the sample size of the literature was not large enough and the representativeness was not strong enough. Third, the accuracy of meta-analysis results may be reduced because subjective factors from researchers cannot be excluded. Fourth, the accuracy and reliability of the literature results were affected by the failure to blind the assessors or the lack of reporting of adverse effects and recurrence rates in the included studies. Fifth, in terms of safety evaluation, most studies did not report adverse effects and could not determine the safety of treatment.

### 4.6. Implications for future studies

More rigorous randomized controlled trials are needed to provide evidence on the effect of TCE on outcomes in people who are overweight or obese. Interventions are needed for TCE with different frequencies, times, and durations to help compare and decide on the best TCE regimen for overweight or obese patients. In addition, the outcomes of the intervention may include not only post-intervention outcomes, but also medium- and long-term outcomes to give a fuller picture of the effect. There is also a need for prespecified protocols with clear reporting, particularly regarding the randomization process, how to manage missing data, and how to prevent deviations from expected interventions in cases where blinding of participants cannot be made due to the nature of the study.

## 5. Conclusion

Our meta-analysis shows that TCE intervention is a beneficial non-pharmacological approach to improving anthropometric and biochemical indicators in overweight and obese subjects, especially in BMI, W, FP, TG, and LDL. The clinical relevance of our findings is pending more extensive trials and more rigorous study designs to strengthen the evidence.

## Author contributions

**Data curation:** Qianfang Yang, Limin Pan.

**Formal analysis:** Qianfang Yang.

**Funding acquisition:** Ting Ye.

**Investigation:** Fan Wang.

**Methodology:** Limin Pan.

**Project administration:** Ting Ye.

**Resources:** Limin Pan.

**Software:** Fan Wang.

**Writing – original draft:** Qianfang Yang.

**Writing – review & editing:** Qianfang Yang.

## Supplementary Material




