# Supplementary material for: Efficacy of traditional Chinese exercises in improving anthropometric and biochemical indicators in overweight and obese subjects: A systematic review and meta-analysis
Source: Medicine (Baltimore). 2023 Mar 24;102(12):e33051. doi: 10.1097/MD.0000000000033051 (PMC10036064; doi:10.1097/MD.0000000000033051)
Supplement: Supplementary file 1 [file medi-102-e33051-s001.pdf]

Supplementary Material

Supplementary Appendix – Electronic searches

Pubmed

("Traditional Chinese Exercise" [Title/Abstract] OR ("Tai-ji" [MeSH Terms] OR "Tai-ji" [Title/Abstract] OR "tai chi" [Title/Abstract] OR "chi tai" [Title/Abstract] OR "tai ji quan" [Title/Abstract] OR "ji quan tai" [Title/Abstract] OR "quan tai ji" [Title/Abstract] OR "Taiji" [Title/Abstract] OR "Taijiquan" [Title/Abstract] OR "t'ai chi" [Title/Abstract] OR "tai chi chuan" [Title/Abstract] OR ("qigong" [MeSH Terms] OR "qi gong" [Title/Abstract] OR "ch'i kung" [Title/Abstract] OR "Yijinjing" [Title/Abstract] OR "Baduanjin" [Title/Abstract] OR "Wuqinxi" [Title/Abstract]) AND ("Obesity" [Mesh Terms] OR "Obesity Disease" [Title/Abstract] OR "simple obesity" [Title/Abstract] OR "adiposis" [Title/Abstract] OR "adiposis fat" [Title/Abstract] OR "fatness" [Title/Abstract] OR "obeseness" [Title/Abstract] OR "obese" [Title/Abstract] OR "morbid obesity" [Title/Abstract] OR "metabolism obesity" [Title/Abstract]) AND ("randomized controlled trials" [Title/Abstract] OR "clinical trial" [Title/Abstract] OR "random allocation" [Title/Abstract] OR "double-blind" [Title/Abstract] OR "randomly" [Title/Abstract] OR "randomization" [Title/Abstract] OR "randomized" [Title/Abstract] OR "RCT" [Title/Abstract] OR "random" [Title/Abstract])

Cochrane library

(((Traditional Chinese Exercise):ti,ab OR (Tai Ji)MeSH OR (Tai-Ji):ti,ab OR (Tai Chi):ti,ab OR (Chi Tai):ti,ab OR (Tai Ji Quan):ti,ab OR (Quan Tai Ji):ti,ab OR (Ji Quan Tai):ti,ab OR (Taiji):ti,ab OR (Taijiquan):ti,ab OR (T'ai Chi):ti,ab OR (Tai Chi Chuan):ti,ab OR (Qigong)MeSH OR (Qi Gong):ti,ab OR (Chi Kung):ti,ab OR (yijinjing):ti,ab OR (baduanjin):ti,ab OR (wuqinxi):ti,ab) AND ((Obesity):ab,ti OR (Obesity Disease):ab,ti OR (simple obesity):ab,ti OR (adiposis):ab,ti OR (adiposis fat):ab,ti OR (fatness):ab,ti OR (obeseness):ab,ti OR (obese):ab,ti OR (morbid obesity):ab,ti OR (metabolism obesity):ab,ti AND ((randomized controlled trials):ti,ab, OR (clinical trial):ti,ab, OR (random allocation):ti,ab OR (double-blind):ti,ab OR (randomly):ti,ab OR (randomized):ti,ab OR (RCT):ti,ab OR (random):ti,ab))

Embase

#1 (("Traditional Chinese Exercise":ab,ti OR "Tai Ji":ab,ti OR "Tai-Ji":ab,ti OR "Tai Chi":ab,ti OR "Chi Tai":ab,ti OR "Tai Ji Quan":ab,ti OR "Quan Tai Ji":ab,ti OR "Ji Quan Tai":ab,ti OR "Taiji":ab,ti OR "Taijiquan":ab,ti OR "Tai Chi Chuan":ab,ti OR "Qigong":ab,ti OR "Qi Gong":ab,ti OR "Chi Kung":ab,ti OR "yijinjing":ab,ti OR "baduanjin":ab,ti OR "wuqinxi":ab,ti)

#2 ("Obesity":ab,ti OR "Obesity Disease":ab,ti OR "simple obesity":ab,ti OR "adiposis":ab,ti OR "adiposis fat":ab,ti OR "fatness":ab,ti OR "obeseness":ab,ti OR "obese":ab,ti OR "morbid obesity":ab,ti OR "metabolism obesity":ab,ti)
#3 ("randomized controlled trials":ab,ti OR "clinical trial" OR "random allocation":ab,ti OR "double-blind":ab,ti OR "randomly":ab,ti OR "randomization":ab,ti OR "randomized":ab,ti OR "RCT":ab,ti OR "random":ab,ti))
#4 #1 AND #2 AND #3

Web of Science

((TI= (Traditional Chinese Exercise OR Tai Ji OR Tai-ji OR Tai Chi OR Chi Tai OR Tai Ji Quan OR Quan Tai Ji OR Ji Quan Tai OR Taiji OR Taijiquan OR T'ai Chi OR Tai Chi Chuan OR Qigong OR Qi Gong OR Chi Kung OR yijinjing OR baduanjin OR wuqinxi) OR AB= (Traditional Chinese Exercise OR Tai Ji OR Tai-ji OR Tai Chi OR Chi Tai OR Tai Ji Quan OR Quan Tai Ji OR Ji Quan Tai OR Taiji OR Taijiquan OR T'ai Chi OR Tai Chi Chuan OR Qigong OR Qi Gong OR Chi Kung OR yijinjing OR baduanjin OR wuqinxi)) AND (TI=((Obesity OR Obesity Disease OR simple obesity OR adiposis OR adiposis fat OR fatness OR obeseness OR obese OR morbid obesity OR metabolism obesity) OR AB=(Obesity OR Obesity Disease OR simple obesity OR adiposis OR adiposis fat OR fatness OR obeseness OR obese OR morbid obesity OR metabolism obesity)) AND (TI=(randomized controlled trials OR clinical trial OR random allocation OR double-blind OR randomly OR randomization OR randomized OR RCT OR random)))

Scopus

#1 TITLE-ABS-KEY ("Traditional Chinese Exercise" OR "Tai-ji" OR "Tai Chi" OR "Chi, Tai" OR "Tai Ji Quan" OR "Ji Quan, Tai" OR "Quan, Tai Ji" OR "Taiji" OR "Taijiquan" OR "T'ai Chi" OR "Tai Chi Chuan" OR "Qi gong" OR "Ch'i Kung" OR "Yijinjing" OR "Baduanjin" OR "Wuqinxi")
#2 TITLE-ABS-KEY ("Obesity" OR "Obesity Disease" OR "simple obesity" OR "adiposis" OR "adiposis fat" OR "fatness" OR "obeseness" OR "obese" OR "morbid obesity" OR "metabolism obesity")
#3 TITLE-ABS-KEY ("randomized controlled trials" OR "clinical trial" OR "random allocation" OR "double-blind" OR "randomly" OR "randomization" OR "randomized" OR "RCT" OR "random")
#4 #1 AND #2 AND #3
