# Supplementary material for: Efficacy of traditional Chinese exercises in improving anthropometric and biochemical indicators in overweight and obese subjects: A systematic review and meta-analysis
Source: Medicine (Baltimore). 2023 Mar 24;102(12):e33051. doi: 10.1097/MD.0000000000033051 (PMC10036064; doi:10.1097/MD.0000000000033051)
Supplement: Supplementary file 2 [file medi-102-e33051-s002.pdf]

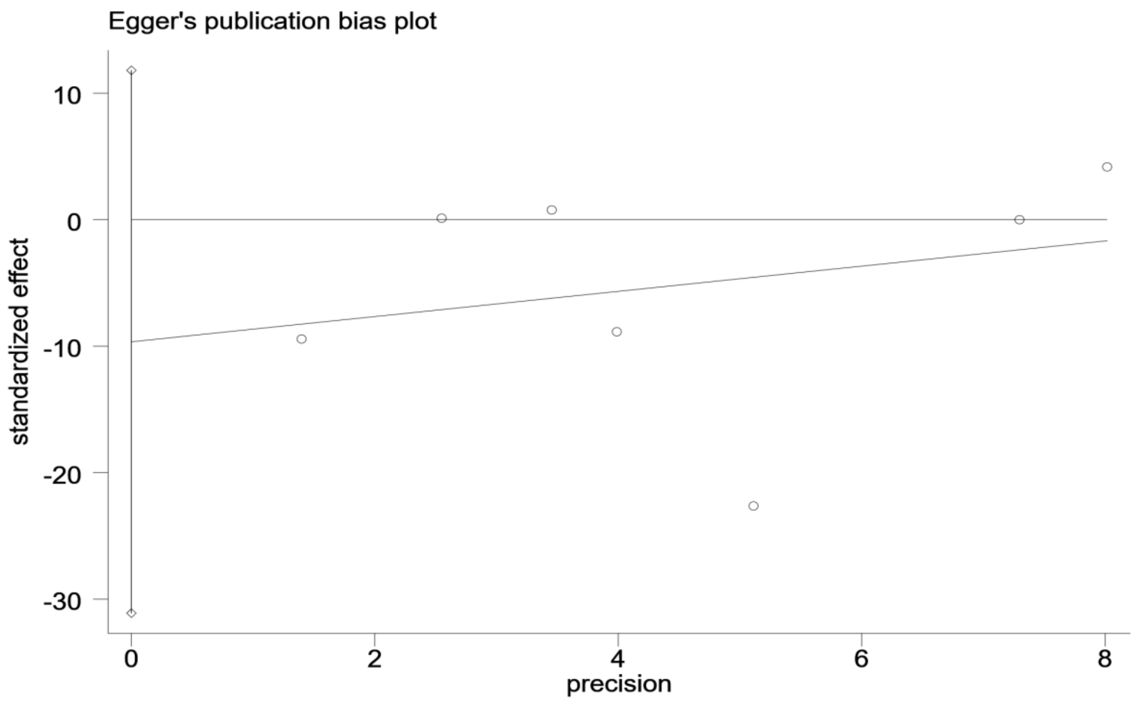

Supplementary Figure 1. Egger’s regression test estimating publication bias for BMI, and the Egger’ test was not statistically significant, suggesting an absence of publication bias (*P*=0.3).

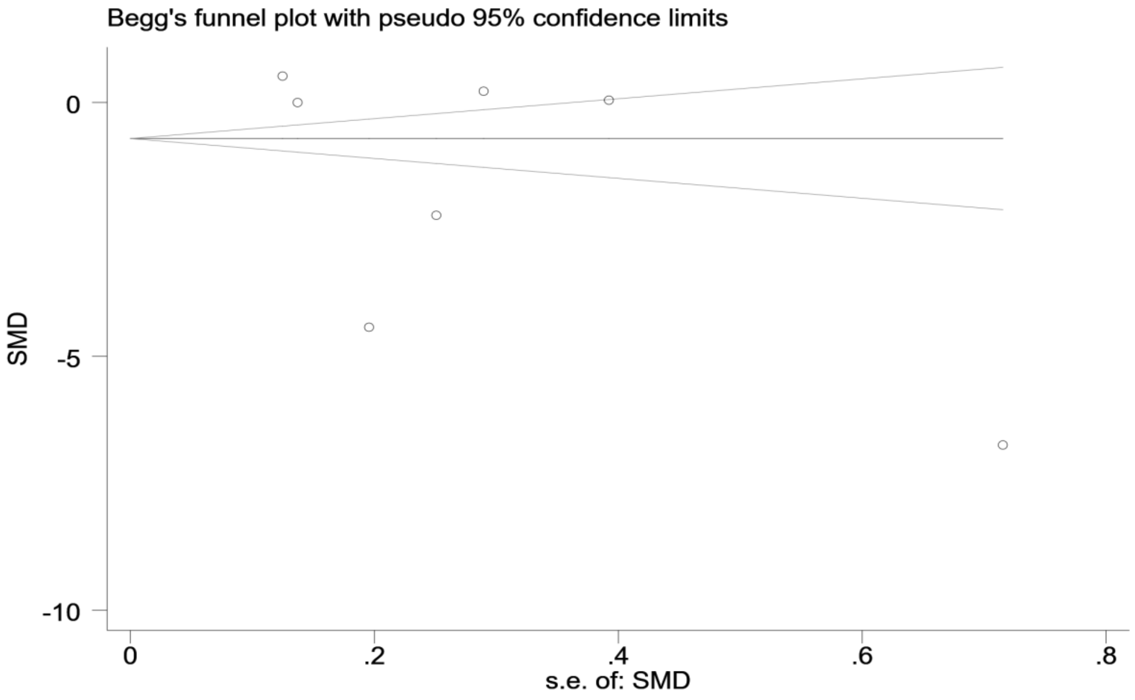

Supplementary Figure 2. Begg’s regression test estimating publication bias for BMI, and the Begg’s test was not statistically significant, suggesting an absence of publication bias (*P*=0.23).

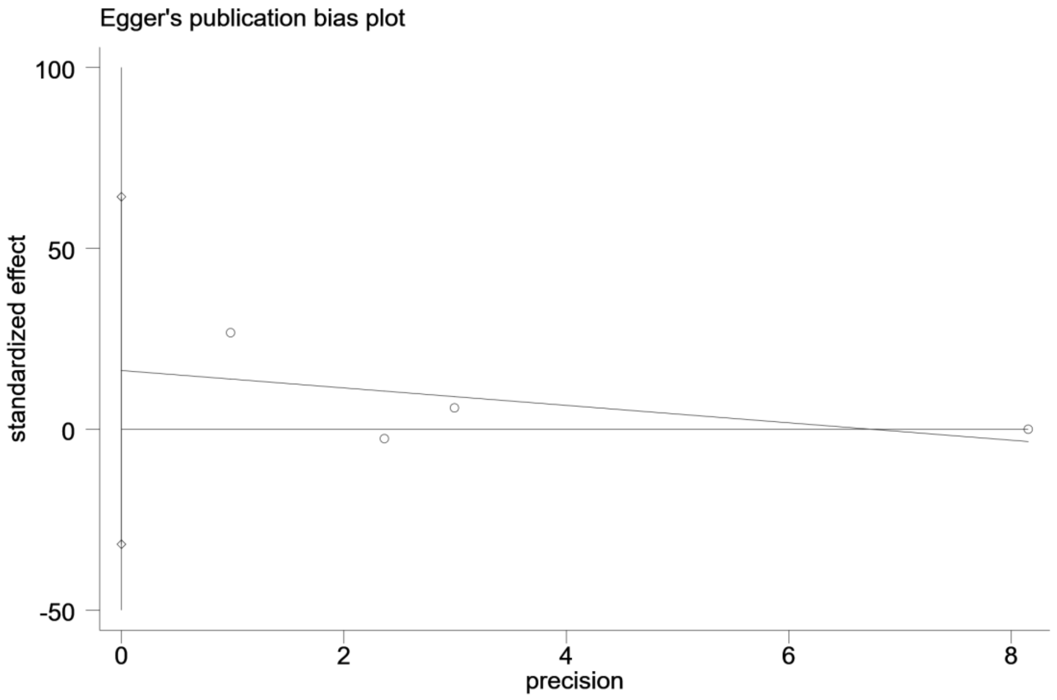

Supplementary Figure 3. Egger’s regression test estimating publication bias for WC, and the Egger’s test was not statistically significant, suggesting an absence of publication bias (*P*=0.18).

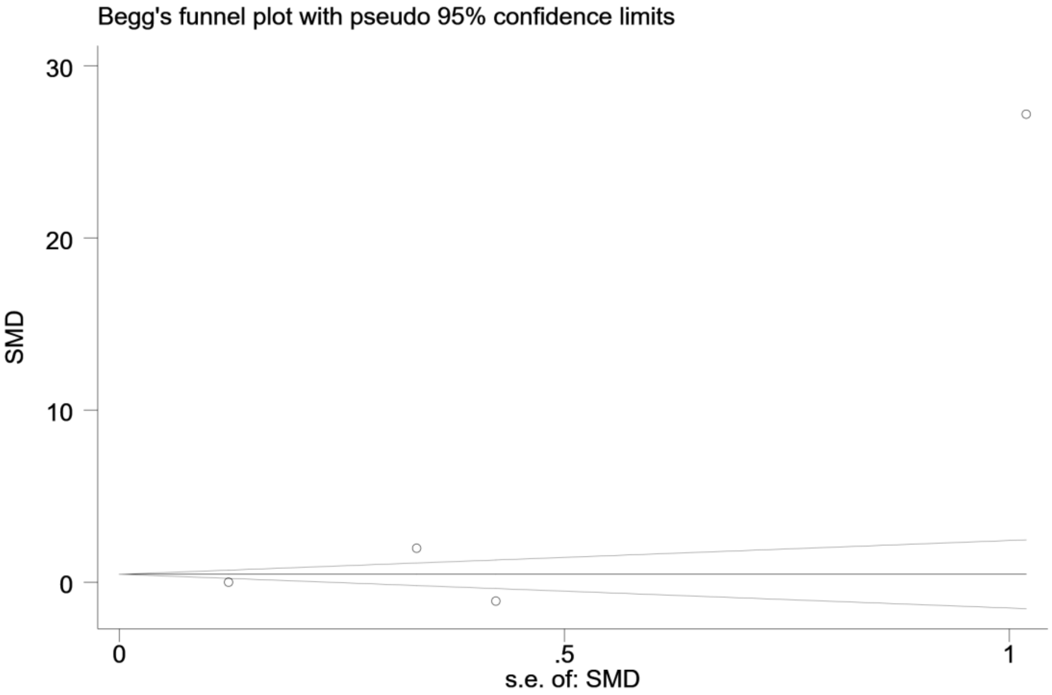

Supplementary Figure 4. Begg's regression test estimating publication bias for WC, and the Begg's test was not statistically significant, suggesting an absence of publication bias (*P*=0.22).

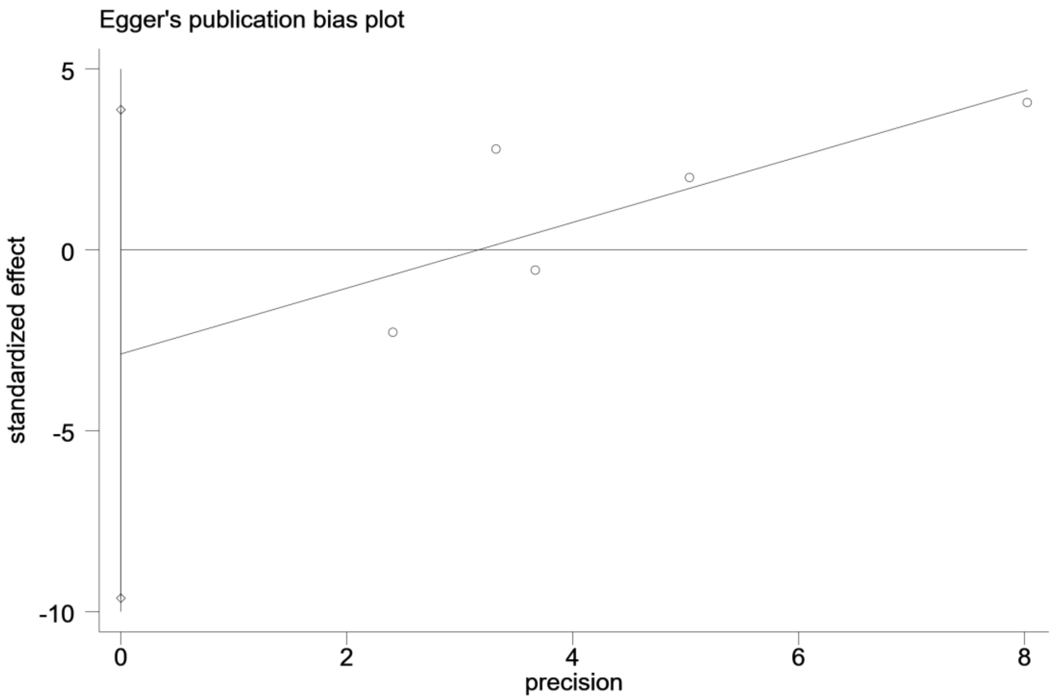

Supplementary Figure 5. Egger's regression test estimating publication bias for TC, and the Egger's test was not statistically significant, suggesting an absence of publication bias (*P*=0.27).

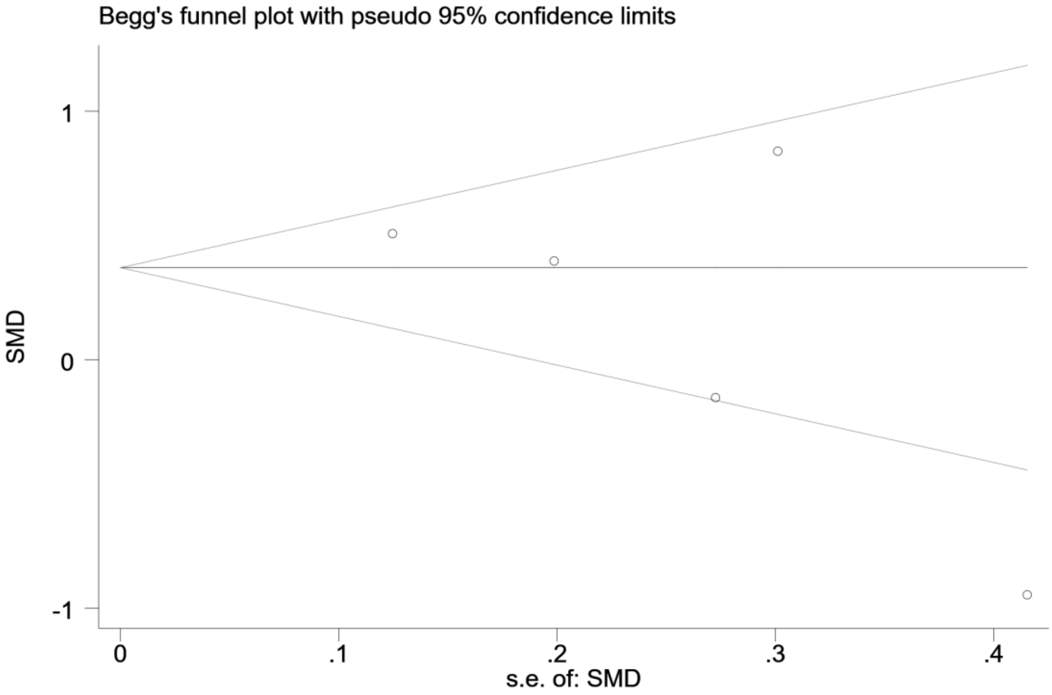

Supplementary Figure 6. Begg’s regression test estimating publication bias for TC, and the Begg’s test was not statistically significant, suggesting an absence of publication bias (*P*=0.46).

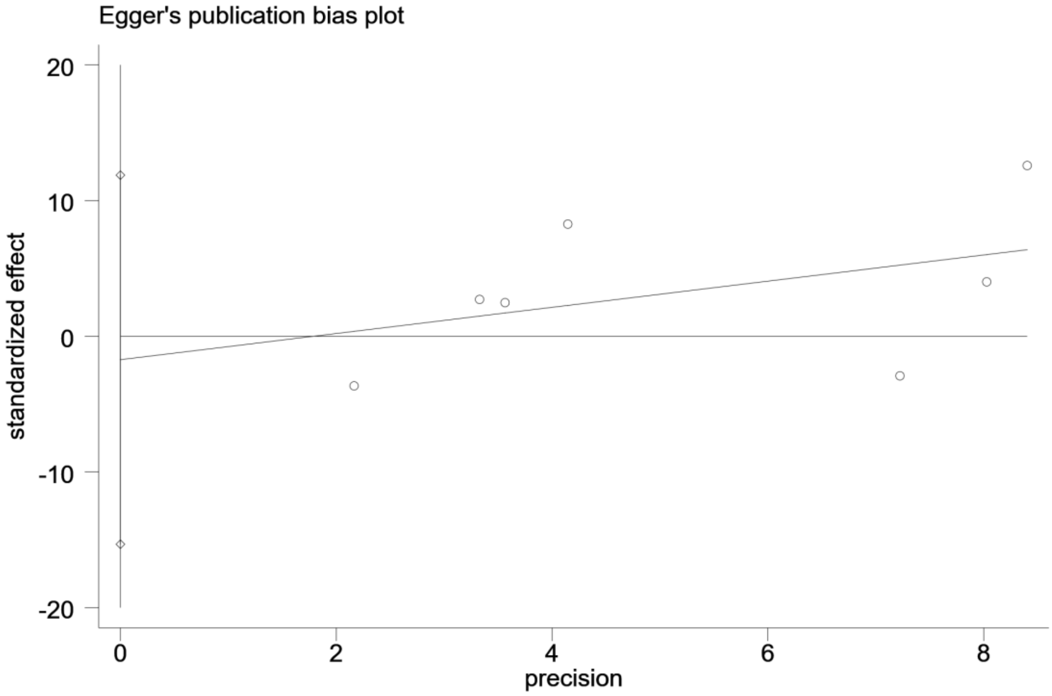

Supplementary Figure 7. Egger's regression test estimating publication bias for TG, and the Egger's test was not statistically significant, suggesting an absence of publication bias (*P*=0.76).

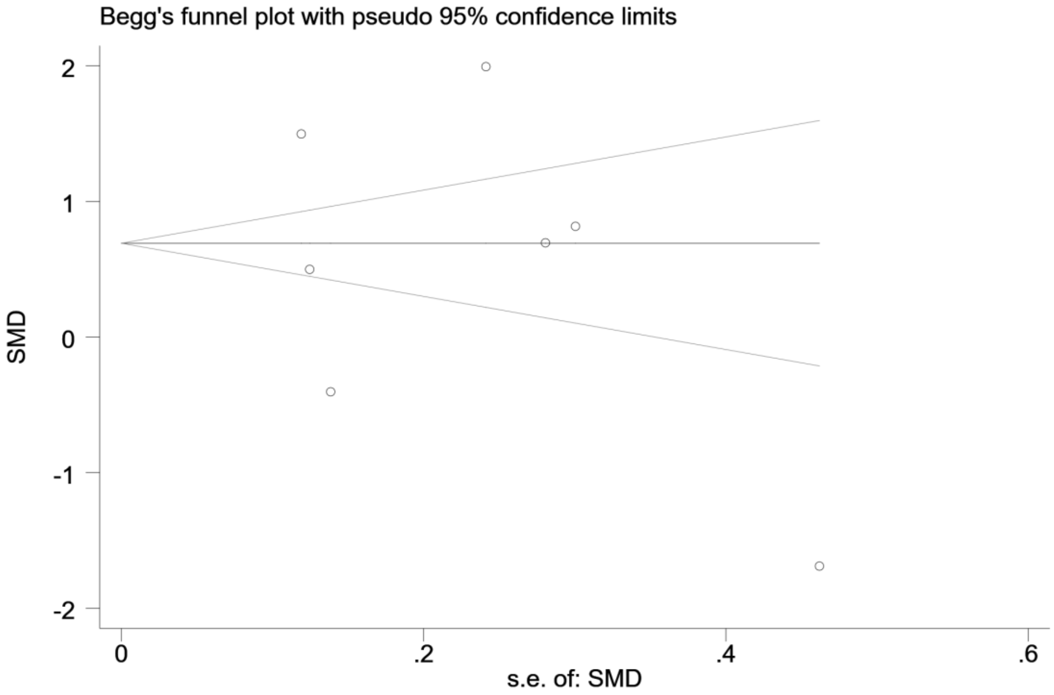

Supplementary Figure 8. Begg's regression test estimating publication bias for TG, and the Begg's test was not statistically significant, suggesting an absence of publication bias (*P*=0.55).
